# Enhancing the Luminescence of La_3_Mg_2_NbO_9_:Mn^4+^ Phosphor through H_3_BO_3_ and Charge Compensator Co-Doping for Use in Plant Growth Lamps

**DOI:** 10.3390/molecules29061402

**Published:** 2024-03-21

**Authors:** Zaifa Yang, Ruoxuan Wang, Shuyu Yang, Hongxia Bu, Jingfen Zhao

**Affiliations:** College of Physics and Electronic Engineering, Qilu Normal University, Jinan 250200, China; wangruoxuan0029@163.com (R.W.); ysy05300205@163.com (S.Y.); buhx666@163.com (H.B.); jingfenzhao@163.com (J.Z.)

**Keywords:** phosphor, charge compensator, H_3_BO_3_, plant growth lamp

## Abstract

Mn^4+^-doped red-light-emitting phosphors have become a research hotspot that can effectively enhance photosynthesis and promote morphogenesis in plants. Herein, the red phosphor La_3_Mg_2_NbO_9_:Mn^4+^ was synthesized through the solid-state reaction method. The effects of adding H_3_BO_3_ and a charge compensator R^+^ (R = Li, Na, K) on the crystal structure, morphology, quantum efficiency, and luminous performance of the La_3_Mg_2_NbO_9_:Mn^4+^ phosphor were systematically analyzed, respectively. The results showed that adding H_3_BO_3_ flux and a charge compensator improved the quantum efficiency and luminescence intensity. The emission intensity of the phosphor was enhanced about 5.9 times when Li^+^ was used as the charge compensator, while it was enhanced about 240% with the addition of H_3_BO_3_ flux. Remarkably, it was also found that the addition of H_3_BO_3_ flux and a charge compensator simultaneously improved the thermal stability at 423 K from 47.3% to 68.9%. The prototype red LED fabricated using the La_3_Mg_2_NbO_9_:Mn^4+^,H_3_BO_3_,Li^+^ phosphor exhibited a perfect overlap with the phytochrome absorption band for plant growth. All of these results indicate that the La_3_Mg_2_NbO_9_:Mn^4+^,H_3_BO_3_,Li^+^ phosphor has great potential for use in agricultural plant lighting.

## 1. Introduction

Fluorescent conversion white-light-emitting diodes (WLEDs) are rapidly replacing traditional illuminant sources with their superior intense brightness, longevity, energy-saving properties, environmental protection, and simple processes, and are gradually becoming mainstream products in the lighting market [1,2,3,4]. Currently, the mainstream WLEDs in the market obtain white light by compounding Y_3_Al_5_O_12_:Ce^3+^ yellow phosphor with InGaN blue LED chips [5,6]. Nevertheless, it is hard for them to satisfy the basic needs of high-quality indoor lighting and backlighting display due to their shortage of red light. Complementing the red emission by adding red phosphor is an effective way to solve the above problems. At present, there are two main types of commercialized red phosphors for WLEDs: Eu^2+^-activated nitride red phosphors, represented by CaAlSiN_3_:Eu^2+^, and Mn^4+^-activated fluoride red phosphors, represented by K_2_SiF_6_:Mn^4+^ [7,8]. However, both of these are limited in their widespread application due to the decrease in quantum efficiency caused by the partial overlap of their excessively broad absorption bands with the emission peaks of the yellow phosphor, as well as the high costs of their synthesis processes due to the need for high-temperature and high-pressure conditions [9,10,11]. Therefore, finding a high-efficiency red phosphor is a top-priority mission nowadays.

Light plays a pivotal role in plant growth, wherein the essential light requirements in the blue (400–480 nm), red (600–680 nm), and far-red (680–780 nm) regions are responsible for photosynthesis, phototropism, and photomorphogenesis, respectively [12,13]. As a transition metal ion, Mn^4+^ has plentiful reserves and a low price compared with traditional rare-earth luminescence centers [14]. In a suitable crystal structure, Mn^4+^ ions with a 3d^3^ electron configuration can emit bright light, and the emission spectrum is mainly concentrated in the red/far-red wavelength range of 620–740 nm [15,16]. Compared with fluoride phosphors, Mn^4+^-activated oxide phosphors with better chemical stability that are synthesized without the highly corrosive HF have drawn much attention in terms of WLEDs, plant illumination, and infrared illumination [17]. To realize a multi-functional Mn^4+^ phosphor, the choice of an optimum host material, which immediately decides the external electric fields, luminescent properties, and thermal stability of the synthetic phosphor, is essential. Recently, niobate has aroused the enthusiasm of researchers in the fields of luminescence, photocatalysis, and optical information storage. Jeong Seog Kim et al. (1999) reported the perovskite-type structure La_3_Mg_2_NbO_9_ (LMN), which has been widely studied. In this paper, compound LMN was selected as the luminescent host, which can be prepared easily with cheap raw materials including Mg_2_(OH)_2_CO_3_, La_2_O_3_, and Nb_2_O_5_. Compared with traditional aluminates, tellurites, etc., niobates have many advantages, such as their affluent crystalline environment, excellent thermal stability, and environmentally friendly experimental methods [18]. To our knowledge, in all of the published literature, relevant developments in the use of the Mn^4+^-doped LMN phosphor have not yet been studied.

In this work, we designed and synthesized LMN:xMn^4+^, LMN:0.6%Mn^4+^,yH_3_BO_3_ (LMN:Mn^4+^,yB), LMN:0.6%Mn^4+^,9%B,5%Li^+^ (LMN:Mn^4+^,B,Li^+^), LMN:0.6%Mn^4+^,9%B, 5%Na^+^ (LMN:Mn^4+^,B,Na^+^), and LMN:0.6%Mn^4+^,9%B,5%K^+^ (LMN:Mn^4+^,B,K^+^) phosphors and studied in detail the influence of H_3_BO_3_ and charge compensator R^+^ on the crystal structures, morphology, quantum efficiency, and luminescent properties of these LMN:Mn^4+^ phosphors. Adding H_3_BO_3_ flux and a charge compensator improved the quantum efficiency and luminescence intensity. In addition, the thermal stability of the fluorescent materials at 423 K increased simultaneously. Also, we demonstrated the potential of these phosphors to enhance sunlight harvesting by fabricating a prototype red LED. All of these results suggest that the LMN:Mn^4+^,B,Li^+^ phosphor shows great potential for use in the production of agricultural plants.

## 2. Results and Discussion

### 2.1. Structural Property Analysis

**Figure 1:**
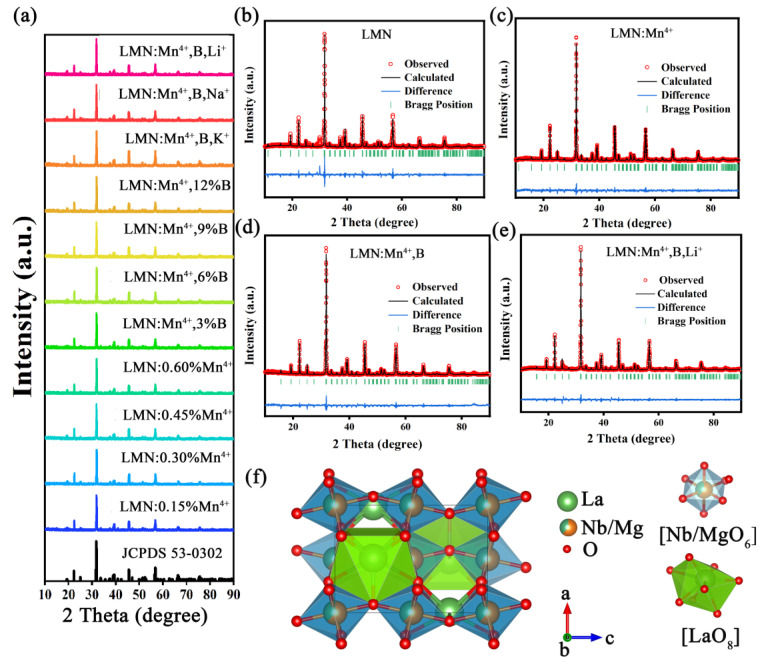
(**a**) XRD patterns of LMN:xMn^4+^, LMN:Mn^4+^,yB, and LMN:Mn^4+^,B,R^+^ (R = Li, Na, K) phosphors. Rietveld refinement profiles for (**b**) LMN; (**c**) LMN:Mn^4+^; (**d**) LMN:Mn^4+^,B; (**e**) LMN:Mn^4+^,B,Li^+^. (**f**) The crystal structure of LMN.

The X-ray powder diffraction (XRD) patterns of LMN:xMn^4+^ and the standard cards (PDF#53–0302) are illustrated in Figure 1a. To analyze the effects of H_3_BO_3_ and the charge compensator on the crystal structure, the phases of the LMN:Mn^4+^,yB (0.03 ≤ y ≤ 0.15) and LMN:Mn^4+^,B,R^+^ (R = Li, Na, K) samples were also tested, as depicted in Figure 1a. All of the characteristic peaks corresponded to the standard cards, indicating that the structural effect of these dopants (Mn^4+^, Li^+^, Na^+^, K^+^, and H_3_BO_3_) on the matrix was not significant. Additionally, the phosphors with H_3_BO_3_ exhibited a higher intensity of XRD patterns, revealing that H_3_BO_3_ can improve the crystallinity of the host. The ionic radii for Mn^4+^ (CN = 6; CN: coordination number), La^3+^ (CN = 8), Mg^2+^ (CN = 6), and Nb^5+^ (CN = 6) are 0.53 Å, 1.16 Å, 0.72 Å, and 0.64 Å, respectively [19]. When introducing Mn^4+^ into the LMN matrix, Mn^4+^ ions preferentially replace Nb^5+^ ions due to the similarity of their ionic radii. In order to obtain further information on the crystal structure of these samples, the LMN, LMN:Mn^4+^, LMN:Mn^4+^,B, and LMN:Mn^4+^,B,Li^+^ samples were subjected to Rietveld refinement by using the general structure analysis system (GSAS) method, as presented in Figure 1b–e. The low residual factors of R_wp_, R_p_, and χ^2^ mean the refinement results are credible [20]. The detailed refined results for LMN, LMN:Mn^4+^, LMN:Mn^4+^,B, and LMN:Mn^4+^,B,Li^+^ are shown in Table 1, which shows that these synthesized samples are classified as the monoclinic system and correspond to the P2_1_/n space group. Furthermore, the cell parameters of LMN remained almost unchanged, except for minor shrinkage after the addition of H_3_BO_3_. According to the refinement results, the crystal structure of LMN is shown in Figure 1f. It is observed that only one La cation site with a Wyckoff position is connected to eight oxygen ions to form the LaO_8_ octahedron. In addition, Mg and Nb at the same site link with six oxygen ions to form the MgO_6_/NbO_6_ polyhedron, which connects with the LaO_8_ polyhedron through sharing the same site. 

**Figure 2:**
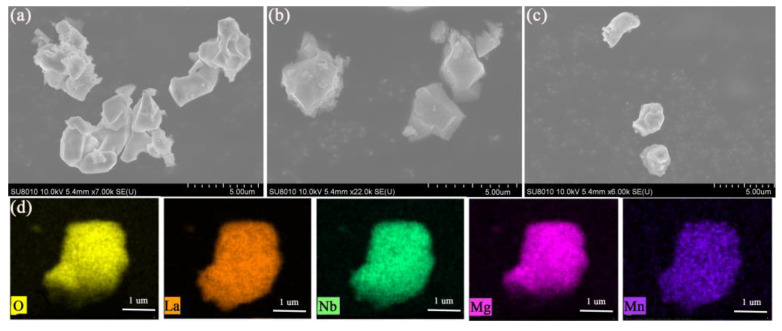
SEM images of (**a**) LMN:Mn^4+^; (**b**) LMN:Mn^4+^,B; and (**c**) LMN:Mn^4+^,B,Li^+^. (**d**) The corresponding element mapping of O, La, Nb, Mg, and Mn in a selected area of the phosphor.

Shape and morphology can have an effect on the performance of luminescent materials. Thus, the scanning electron microscopy (SEM) images of LMN:Mn^4+^, LMN:Mn^4+^,B, and LMN:Mn^4+^,B,Li^+^ are shown in Figure 2a–c. As is obvious, the LMN:Mn^4+^ phosphor shows an irregular polyhedron morphology and micro-granularity with a particle size of approximately 2–5 μm. The surface morphology shows that the grain shape is significantly more anisotropic and less agglomerated after the introduction of the H_3_BO_3_ [21]. The melting of H_3_BO_3_ can enhance the sliding and rotation of the particles, promoting contact between the particles and particle growth and thus increasing the size of the particles [22]. In addition, it is widely known that phosphors with a regular morphology can improve paste properties, improve the filler density, and enhance phosphor luminescence performance [23]. Moreover, the elemental distributions of La, Mg, Nb, O, and Mn shown in Figure 2d clearly show that the constituent elementals are uniformly distributed in the LMN:Mn^4+^,B,Li^+^ powder.

### 2.2. Electronic Properties

**Figure 3:**
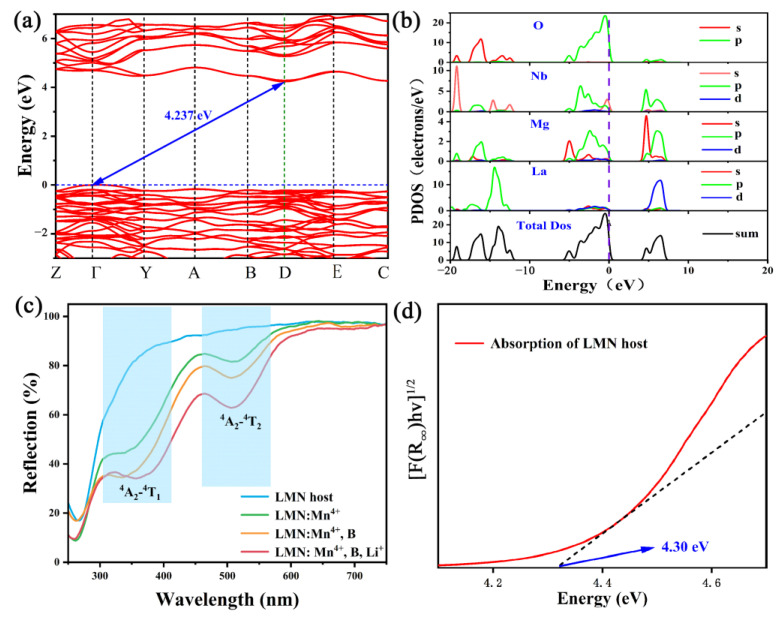
(**a**) Electronic band structure (The red lines represent the band) and (**b**) partial density of states of LMN. (**c**) DRS of LMN host, and LMN:Mn^4+^, LMN:Mn^4+^,B, and LMN:Mn^4+^,B,Li^+^ phosphors. (**d**) The experimental energy gap of the LMN host and the dashed line is the tangent of the curve.

The electronic structural characteristics of matrix materials have a significant impact on luminescent materials. In order to gain insight into its electronic properties, the electronic energy band structure of LMN was studied using the Cambridge Sequential Total Energy Package (CASTEP) module. As shown in Figure 3a, the top of the valence band (VB) located at point Γ compares with the bottom of the conduction band (CB) located at point D. This result indicates that the LMN matrix is an indirect bandgap semiconductor and its energy gap (Eg) is 4.237 eV, which indicates that it is sufficient for the energy level of Mn^4+^ to be the luminescent material [24]. The maps of the partial and total density of states are given in Figure 3b, which can help to understand the band structure component deeply. Compared with other atoms, the p orbital of Nb and O make the most contributions to the CB, which indicates that electronic orbitals within the NbO_6_ octahedron are rather discrete. Thus, the Mn^4+^ ions doped at the Nb^5+^ site would also have a high possibility to generate and obtain free carriers through the CB [25].

Figure 3c depicts the diffuse reflectance spectra (DRS) of the LMN host, LMN:Mn^4+^, LMN:Mn^4+^,B, and LMN:Mn^4+^,B,Li^+^. At 450~800 nm, the LMN presents a high-reflectance plateau, while at 200~450 nm, the reflectance of the LMN decreases sharply because of host absorption. Compared with the host material, the LMN:Mn^4+^, LMN:Mn^4+^,B, and LMN:Mn^4+^,B,Li^+^ samples show more prominent absorption peaks around 300 nm, which resulted from the O^2−^→Mn^4+^ charge transfer band (CTB). Additionally, two wide peaks are observed from 350 to 550 nm, which belong to the ^4^A_2g_→^4^T_1g_ and ^4^A_2g_→^4^T_2g_ transitions of the Mn^4+^ ions, respectively [26]. Furthermore, LMN:Mn^4+^,B,Li^+^ shows the most prominent absorption, followed by the LMN:Mn^4+^,B sample and the LMN:Mn^4+^ sample. Based on the DRS of the LMN host and the theory given by Kubelka–Munk, the E_g_ can be estimated with the following equation [27]:

(1) $${\left[h\nu (R_{\infty })\right]}^{n}=A(h\nu -E_{g})$$

where h, ν, and A denote the absorption factor, Planck constant, and constant, respectively. n = 1/2 or 2 indicates that the sample is an indirect or direct bandgap semiconductor [28]. According to the results in Figure 3a, LMN is an indirect bandgap semiconductor, so the value of n is 1/2. The curve of [hν(R_∞_)]^1/2^ versus the photon energy of the LMN host is shown in Figure 3d, and the calculated value of E_g_ is 4.30 eV, which is close to the result of the theoretical calculation (4.237 eV).

### 2.3. Optical Luminescence Properties of Samples

**Figure 4:**
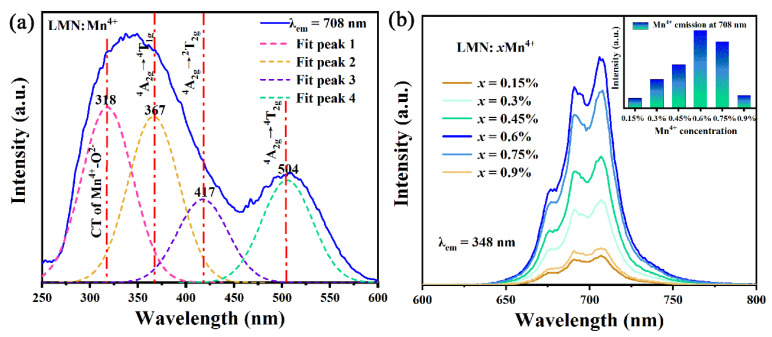
(**a**) The PLE spectra of LMN:Mn^4+^. (**b**) The PL spectra of LMN:xMn^4+^. The inset shows the dependence of the PL intensity on the Mn^4+^ concentration level for the LMN:xMn^4+^ phosphors.

The photoluminescence excitation (PLE) spectra of the LMN:Mn^4+^ phosphor, which are composed of two broad excitation bands in the range of 250–600 nm, are shown in Figure 4a. The one located at 348 nm compares with the other located at 504 nm. In addition, the PLE spectra can be fitted using the Gaussian distribution method to form four characteristic bands peaking around 504, 417, 367, and 318 nm, which can be attributed to ^4^A_2g_→^4^T_2g_, ^4^A_2g_→^2^T_2g_, ^4^A_2g_→^4^T_1g_ transitions of the Mn^4+^ ions and the CTB of O^2−^→Mn^4+^, respectively [29]. As depicted in Figure 4b, the dependence of photoluminescence (PL) spectra for LMN:xMn^4+^ (0.15% ≤ x ≤ 0.9%) is excited at 348 nm. The LMN:xMn^4+^ phosphors exhibit a deep-red luminescence attributed to the ^2^E_g_→^4^A_2g_ spin-forbidden transition of the Mn^4+^ ions centered at 708 nm. Furthermore, as the concentration of the Mn^4+^ ions increases, the PL spectra show the same peak pattern and position except for different intensities. The inset of Figure 4b demonstrates that the luminous intensity ascends and then descends with the doping concentration of Mn^4+^ ions from 0.15% to 0.9%. The optimal luminescence concentration is 0.6% due to the concentration quenching effect [30].

**Figure 5:**
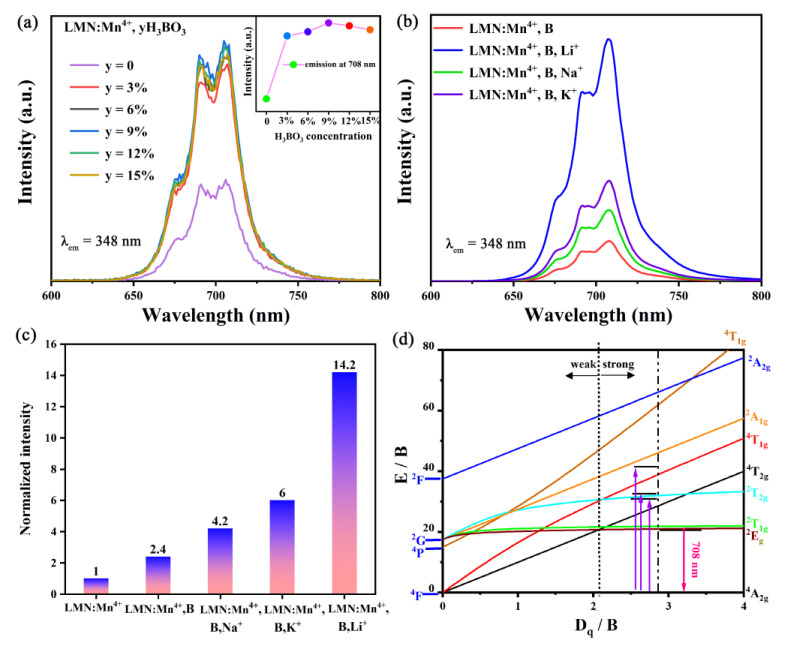
The PL spectra for (**a**) LMN:Mn^4+^,yB and the relative point line diagram; and (**b**) LMN:Mn^4+^,B,R^+^ (R = K, Na, Li) phosphors. (**c**) The normalized luminescence intensity of different phosphors. (**d**) Tanabe–Sugano energy level diagram of a Mn^4+^ ion in the LMN host.

To improve the luminescence properties, the flux of H_3_BO_3_ powders with different doping contents were added to the LMN:Mn^4+^ sample. As shown in Figure 5a, the PL spectra of LMN:Mn^4+^,yH_3_BO_3_ (y = 0, 3%, 6%, 9%, 12%, and 15%) phosphors were systematically studied. Except for the luminous intensity, no notable changes were detected with the concentration of H_3_BO_3_ changing from 3% to 15%. However, compared to the LMN:Mn^4+^ sample, the luminescence intensity of all doped H_3_BO_3_ phosphors significantly improved. Doping with H_3_BO_3_ can reduce the sintering temperature, enhance the crystallinity of the sample, and increase the luminous intensity [31]. The variation in luminescence intensity with different concentrations of H_3_BO_3_ is shown in the illustration of Figure 5a. Furthermore, the best dopant concentration of H_3_BO_3_ was 9%. The LMN:Mn^4+^,B,R^+^ (R = K, Na, Li) phosphors were successfully synthesized, which can assist researchers with exploring the improvement of luminescent properties. As shown in Figure 5b, it is noted that doping with K^+^, Na^+^, and Li^+^ ions enhances the luminous intensity when comparing the PL spectra of LMN:Mn^4+^,B. In our opinion, the main reason for such a result comes from the charge balance. The ionic radii of K^+^, Li^+^, and Na^+^ are similar to that of Nb^5+^, so R^+^ (R = K, Na, Li) will be replaced by Nb^5+^ to form [Mn^4+^-R^+^] pairs. And these [Mn^4+^-R^+^] pairs can interrupt the adverse energy transfer among neighboring Mn^4+^ ions to increase the luminescence intensity [32]. Accordingly, the luminous intensity of LMN:Mn^4+^,B can be enhanced through using K^+^, Na^+^, and Li^+^ co-doping ions to improve the charge compensation. It is noteworthy that when Li+ ions were doped into LMN:Mn^4+^B, the luminescence intensity of the Li^+^ ions was intensified much more than that of the K^+^/Na^+^ ions. These outcomes likely stemmed from the fact that [Mn^4+^-Li^+^] interrupts the energy transfer between Mn^4+^ ions more efficiently than the ionic substitution between K^+^ and Na^+^. Specifically, the luminescence intensity of the LMN:Mn^4+^ sample is considered a regular standard, as shown in Figure 5c, and the luminescent strength of LMN:Mn^4+^,B,Li^+^ can increase up to 1420%, while the LMN:Mn^4+^,B, LMN:Mn^4+^,B,K^+^, and LMN:Mn^4+^,B,Na^+^ samples can increase up to 240%, 420%, and 600%, respectively. The energy level diagram of Mn^4+^ in the octahedral coordination situation is further described using a Tanabe–Sugano diagram. This energy level transition process of LMN:Mn^4+^ is matched well with the above results, as depicted in Figure 5d. In general, the crystal field strength (D_q_) and Racah parameters (B, C) are calculated as follows [33]:

(2) Dq=EA2g4→T2g4/10

(3) $$\frac{D_{q}}{B}=\frac{15(x-8)}{x^{2}-10x}$$

(4) x=E(A2g4→T1g4)−E(A2g4→T2g4)Dq

(5) E(Eg2→A2g4)B=3.05CB+7.9−1.8BDq

According the above equations, the results of D_q_, B, and C values for Mn^4+^ in the LMN are 1984.1, 724.1, and 2911.3 cm^−1^. Generally speaking, when D_q_/B is greater than 2.1, the dopant ions are generally considered to be in a strong crystal field environment [34]. Here, the D_q_/B value is calculated as 2.74, indicating that the Mn^4+^ ions are located in a strong crystal field. By using the same method, the D_q_/B value of LMN:Mn^4+^,B,Li^+^ is calculated to be 2.83. These results demonstrate that the doping of H_3_BO_3_ and Li^+^ into the lattice of the LMN:Mn^4+^ phosphor can efficiently enhance the crystal field strength of Mn^4+^.

**Figure 6:**
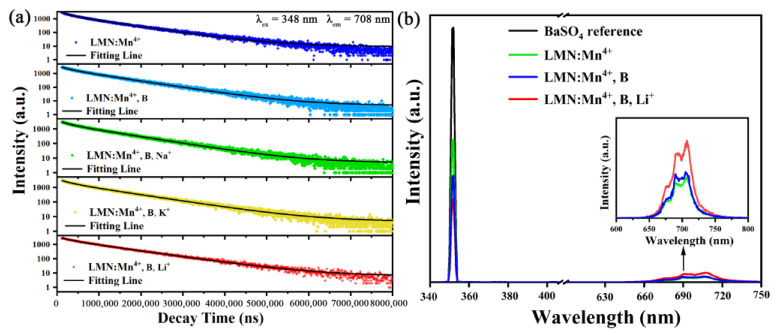
(**a**) Luminescence decay curves of LMN:Mn^4+^, LMN:Mn^4+^,B, and LMN:Mn^4+^,B,R^+^ (R = Li, Na, K) phosphors. (**b**) Excitation line of BaSO4 and the emission spectra of LMN:Mn^4+^, LMN:Mn^4+^,B, and LMN:Mn^4+^,B,Li^+^ phosphors.

To further investigate the effects of H_3_BO_3_ and the charge compensator, decay curves of the LMN:Mn^4+^, LMN:Mn^4+^,B, and LMN:Mn^4+^,B,R^+^ (R = K, Na, Li) phosphors were obtained by monitoring at 708 nm, as depicted in Figure 6a. The experimental data of the decay curves were fitted using the following two-exponential function [35]:

(6) $$I=I_{0}+A_{1}\exp(-t/\tau_{1})+A_{2}\exp(-t/\tau_{2})$$

where I_(t)_ and I_0_ are the intensities at time t and t = 0, respectively. A_1_ and A_2_ are constants, and τ_1_ and τ_2_ are the fast and slow lifetimes, respectively. Based on these fitting parameters, the average lifetime τ can be estimated using the following formula:

(7) $$\tau =({A_{1}\tau_{1}}^{2}+{A_{2}\tau_{2}}^{2})/(A_{1}\tau_{1}+A_{2}\tau_{2})$$

Therefore, the average luminescence lifetimes of LMN:Mn^4+^, LMN:Mn^4+^,B, and LMN:Mn^4+^,B,R^+^ (R = K, Na, Li) are evaluated to be 0.847 ms, 0.863 ms, 0.901 ms, 0.883 ms, and 0.975 ms, as depicted in Table 2. Based on the XRD structural analysis, lattice shrinkage also solidifies the structural rigidity, thereby inhibiting non-radiative transitions [36]. The results show that a more efficient fluorescence lifetime can be acquired from adding H_3_BO_3_ into LMN:Mn^4+^. Additionally, the lifetimes of LMN:Mn^4+^,B,R^+^ (R = K, Na, Li) are similar and larger than those of LMN:Mn^4+^,B, suggesting that the doping of R^+^ (R = K, Na, Li) ions can efficiently decrease the feasibility of non-radiative transitions of Mn^4+^ ions.

In addition, the internal quantum efficiency (IQE) levels of the LMN:Mn^4+^, LMN:Mn^4+^,B, and LMN:Mn^4+^,B,Li^+^ phosphors were measured and are shown in Figure 6b and Table 2. The IQE value and absorption coefficient (α abs) can be acquired using the following equation [37]:

(8) $$\eta =\frac{\int L_{s}}{\int E_{R}-\int E_{s}}$$

(9) $$\alpha_{abs}=\frac{\int E_{R}-\int E_{s}}{\int E_{R}}$$

where L_S_ is the spectral intensity of PL, E_S_ is the spectral intensity of the PLE with the sample, and E_R_ is the spectral intensity of the PLE without it in the integrating sphere. Figure 6b shows that the α abs values of the LMN:Mn^4+^, LMN:Mn^4+^,B, and LMN:Mn^4+^,B,Li^+^ phosphors were computed to be 44.2%, 58.5%, and 67.3%. Meanwhile, the IQE values of the LMN:Mn^4+^ phosphors were improved remarkably from 43.3% to 50.2% for LMN:Mn^4+^,B and 61.7% for LMN:Mn^4+^,B,Li^+^. Compared with the LMN:Mn^4+^ phosphor, LMN:Mn^4+^,B and LMN:Mn^4+^,B,Li^+^ had a higher quantum yield, which was mainly due to the strong absorption of the phosphors to the 348 nm band. In addition, the obtained results are superior to that for several red phosphors including Li_2_MgTiO_4_:Mn^4+^ (IQE = 32%) [29], Li_6_SrLa_2_Sb_2_O_12_:Mn^4+^ (IQE = 17%) [33], Sr_3_NaSbO_6_:Mn^4+^ (IQE = 56.2%) [38], Sr_3_LiSbO_6_:Mn^4+^ (IQE = 52.3%) [39], SrAl_3_BO_7_:Mn^4+^ (IQE = 32%) [40], and Ba_2_SrWO_6_:Mn^4+^ (IQE = 51.5%) [41]. According to these excellent results from the LMN:Mn^4+^,B,Li^+^ sample, this phosphor shows great possibilities for practical applications in LEDs.

### 2.4. Thermal Stability Analysis of Phosphors

**Figure 7:**
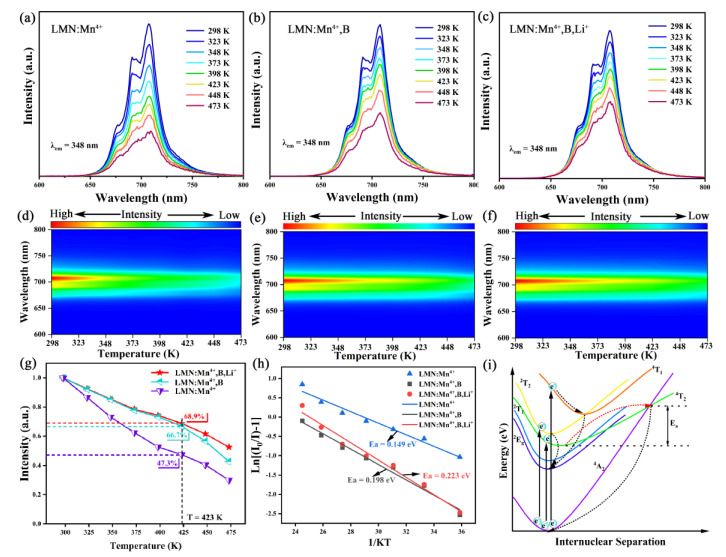
Temperature-dependent PL spectra and the corresponding contour maps of the thermal evolution of (**a**,**d**) LMN:Mn^4+^, (**b**,**e**) LMN:Mn^4+^,B, and (**c**,**f**) LMN:Mn^4+^,B,Li^+^. (**g**) Relative integrated intensity as a function of the temperature. (**h**) Thermal quenching activation energy of these phosphors. (**i**) Configurational coordinate diagram for the Mn^4+^ ions (The black dashed arrow represents the transition process generated by heating and the red dashed arrow represents the non-radiative transition).

An important indicator of phosphor commercialization potential is thermal stability. Subsequently, Figure 7a–c show the thermal quenching of LMN:Mn^4+^, LMN:Mn^4+^,B, and LMN:Mn^4+^,B,Li^+^ and their emission spectra at higher temperatures. In addition, Figure 7d–f display the corresponding contour maps of the thermal evolution of these samples excited at 348 nm from 298 K to 473 K. The relevant emission intensity of these samples exhibits a typical decreasing trend with the increase in temperature. This phenomenon is consistent with the thermal quenching effect found in most oxide phosphor systems doped with Mn^4+^ [42]. Figure 7g depicts that the luminescence intensity of LMN:Mn^4+^, LMN:Mn^4+^,B, and LMN:Mn^4+^,B,Li^+^ can maintain 47.3%, 66.7%, and 68.9% of the initial intensity at 423 K. The results show that the doping of H_3_BO_3_ and Li^+^ ions significantly improved the thermal stability of the materials, which further demonstrates the effectiveness of the above-described H_3_BO_3_ and Li^+^ ion doping in reducing the probability of non-radiative transitions [43]. As shown in Table 3, the results from the LMN:Mn^4+^,B,Li^+^ sample display greater thermal stability compared to some other up-to-date studies of Mn^4+^-doped oxide phosphors, such as Sr_2_LuTaO_6_:Mn^4+^ (I_423 K_/I_298 K_ = 25%) [9], SrLa_2_Al_2_O_7_:Mn^4+^ (I_423 K_/I_298 K_ = 43%) [34], CaY_0.5_Ta_0.5_O_3_:Mn^4+^ (I_423 K_/I_298 K_ = 50%) [44], CaLaLiTeO_6_:Mn^4+^ (I_423 K_/I_298 K_ = 63%) [45], Mg_3_Ga_2_SnO_8_:Mn^4+^ (I_423 K_/I_298 K_ = 50%) [46], Cs_2_NbOF_5_:Mn^4+^ [47], and Li_6_SrLa_2_Sb2O_12_: Mn^4+^ [48].

In general, the activation energy (E_a_) is the distance from the bottom of the ^4^T_2_ energy level to the intersection of the ^4^T_2_ and ^4^A_2_ energy levels [49]. Figure 7i illustrates that, in the conformational coordinate scheme, the ^4^T_2_ and ^4^A_2_ energy levels intersect the activation energy. As the temperature increases, electrons situated in the ^4^T_2_ energy level cross the energy barrier of the activation energy to jump to the ^4^A_2_ energy level and make a non-radiative transition to the ground, resulting in luminescence quenching [50]. The E_a_ is calculated using the following Arrhenius equation for further analysis of the thermal quenching effect [51]:

(10) $$I_{T}=\frac{I_{0}}{1+c\exp[-(E_{a}/KT)]}$$

Using this Arrhenius formula, the activation energies were calculated for each sample. The results were 0.149 eV for LMN:Mn^4+^, 0.198 eV for LMN:Mn^4+^,B, and 0.223 eV for LMN:Mn^4+^,B,Li^+^, as depicted in Figure 7h. Apparently, the larger the E_a_, the better the thermal stability of the phosphors.

### 2.5. Potential Applications

**Figure 8:**
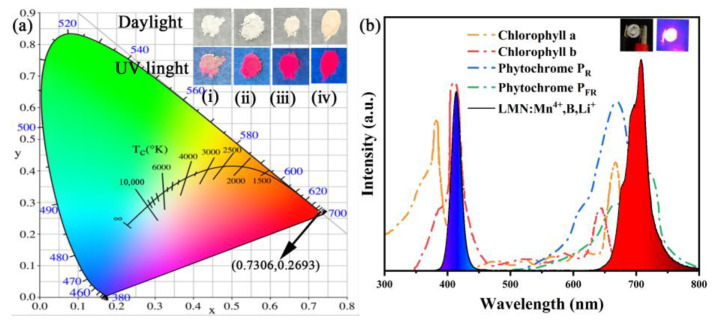
(**a**) CIE chromaticity coordinates diagram of (ⅰ) the LMN host and (ⅱ) LMN:Mn^4+^, (ⅲ) LMN:Mn^4+^,B, and (ⅳ) LMN:Mn^4+^,B,Li^+^ phosphors; (**b**) EL spectrum of the red LED device driven by a 410 nm chip compared with the absorption spectra of plants to natural light.

The color coordinate is an important index for fluorescent materials. Based on the results of emission spectrum data excited at 348 nm, the Commission International deI’Eclairage (CIE) color coordinates for the LMN:Mn^4+^,B,Li^+^ phosphor were calculated to be (0.7306, 0.2693). Figure 8a shows that CIE chromaticity coordinates are located in the deep-red region and the coordinates are located at the edge of the CIE plot, indicating that this phosphor has a high color purity. The inset of Figure 8a shows the electronic photographs of the non-doped LMN host and the LMN:Mn^4+^, LMN:Mn^4+^,B, and LMN:Mn^4+^,B,Li^+^ red phosphors exposed to daylight and 365 nm UV light. Compared to the matrix material, the deep-red emission from the other samples under 365 nm UV lamps can be clearly observed with the naked eye, and LMN:Mn^4+^,B,Li^+^ shows the strongest luminescence intensity. This result indicates that the LMN:Mn^4+^,B,Li^+^ phosphor as a red-light-emitting phosphor for WLEDs shows good prospects. For evaluating the availability of the LMN:Mn^4+^,B,Li^+^ phosphor in practical applications, we successfully combined this phosphor with a 410 nm chip to fabricate an LED. Figure 8b depicts the electroluminescence (EL) spectrum of the LED driven at a current of 20 mA. It can be observed that the PL spectrum contains two broadband peaks: a red emission band located in the 600–750 nm range and a blue emission band centered at 410 nm. The inset of Figure 8b also shows that the photo of the LED light driven with a 20 mA current illuminates a bright red emission. In plants, the chlorophylls A and B and the phytochromes P_R_ and P_FR_ are vital for plant growth [52]. Figure 8b also shows the absorption spectra of these four major phytochromes. Notably, the PL spectra of the red LED perfectly overlap with the phytochrome absorption band for plant growth. These results confirm that the LMN:Mn^4+^,B,Li^+^ phosphor can be efficiently used to prepare red LEDs for plants.

## 3. Materials and Methods

### 3.1. Preparation of Materials and LEDs

The LMN:xMn^4+^, LMN:Mn^4+^,yB, and LMN:Mn^4+^,B,R^+^ (R = Li, Na, K) phosphors were obtained through the solid-state method. La_2_O_3_ (99.99%), Mg_2_(OH)_2_CO_3_ (A.R. (Analytical Reagent)), Nb_2_O_5_ (A.R.), MnCO_3_ (A.R.), H_3_BO_3_ (A.R.), Li_2_CO_3_ (A.R.), Na_2_CO_3_ (A.R.), and K_2_CO_3_ (A.R.) were the starting materials, purchased from Shanghai Aladdin Biochemical Technology Co., Ltd. (Shanghai, China). The samples were weighed according to the molar ratio of La_2_O_3_:Mg_2_(OH)_2_CO_3_:Nb_2_O_5_ = 3:0.8:1. In addition, H_3_BO_3_ was employed as the cosolvent source and Li_2_CO_3_, Na_2_CO_3_, and K_2_CO_3_ were employed as the charge compensators added into the LMN:xMn^4+^ phosphors. The raw materials were mixed and ground thoroughly for 30 min using an agate mortar. After completely grinding the materials, the obtained mixed powder was transferred to an alumina crucible and heated to 450 °C at a rate of 10 °C/min in an air atmosphere. Then, the evenly mixed materials were moved from the crucible to calcine at 1350 °C for 8 h in a muffle furnace. Eventually, these harvested products could be obtained to make the following characterizations. A red pc-LED device for planting was fabricated using the as-prepared LMN:Mn^4+^,B,Li^+^ phosphors and a 410 nm blue LED chip. Following a typical fabrication process, the phosphors were uniformly mixed with silicone resin A and B (A:B = 1:1) in agate mortar, and the resulting mixture was coated onto LED chips. The packaged device was cured at 75 °C for 6 h to form pc-LED devices.

### 3.2. Characterization of Materials

The crystalline structures of these harvested products could be identified using XRD patterns using a Bruker D8 Advance diffractometer (Billerica, MA, USA) with Cu-Kα (λ = 0.15406 Å) radiation. The morphology and elemental investigations were examined by using scanning electron microscopy (Hitachi S-4800 SEM, Tokyo, Japan). The UV–Vis–NIR DRS were detected through using a Hitachi UV–Vis–NIR spectrophotometer (UH4150). The PL, PLE, temperature-dependent emission spectra, and luminescence decay curves were obtained using an Edinburgh fluorescence spectrophotometer (FLS1000, Livingston, UK) with a 450 W xenon irradiation source. The IQE was tested with the same FLS1000 instrument using an integrating sphere. The Cambridge Sequential Total Energy Package (CASTEP, VASP, Version 6.1.0) was adopted to carry out the density functional theory (DFT) calculations within the generalized gradient approximation. The EL performances of the pc-LED devices were measured using a photoelectric measuring system (HAAS 2000, Hangzhou, China) with an integrating sphere.

## 4. Conclusions

In summary, LMN:xMn^4+^, LMN:Mn^4+^,yB, and LMN:Mn^4+^,B,R^+^ (R = K, Na, Li) phosphors were obtained through a solid-state method. The physicochemical properties of these phosphors were studied in terms of their structural, elemental, morphological, electronic, and optical characteristics. The XRD results showed that LMN:Mn^4+^,B,Li^+^ had a monoclinic structure and corresponded to the P2_1_/n space group. The DRS showed the Eg of LMN, which matched well with the density functional theory. The phosphors exhibited two wide excitation bands from 250 to 600 nm, which originated from the CTB of the O^2−^→Mn^4+^ and ^4^A_2g_→^4^T_2g_, ^4^A_2g_→^2^T_2g_, ^4^A_2g_→^4^T_1g_ transitions in the Mn^4+^ ions. Under 348 nm excitation, the LMN:Mn^4+^ phosphor exhibited a deep-red emission centered at 708 nm because of the ^2^E_g_→^4^A_2g_ transition in the Mn^4+^ ions. To enhance the luminescence intensity of the LMN:Mn^4+^ sample, H_3_BO_3_ and a charge compensator were added, and the results showed that the quantum efficiency and luminescence intensity were improved effectively. In addition, the thermal stability of the LMN:Mn^4+^,B,Li^+^ phosphor had been significantly improved through the doping with H_3_BO_3_ and Li^+^ ions. Impressively, the prototype red LED fabricated with the LMN:Mn^4+^,B,Li^+^ phosphor exhibited perfect overlap with the phytochrome absorption band for plant growth. All in all, these results demonstrate that the LMN:Mn^4+^,B,Li^+^ phosphor can be effectively applied in indoor artificial lighting for plants.

## Figures and Tables

**Table 1:** The final crystallography parameters and detailed refinement results for the LMN, LMN:Mn^4+^, LMN:Mn^4+^,B, and LMN:Mn^4+^,B,Li^+^ samples.

Sample	LMN	LMN:Mn^4+^	LMN:Mn^4+^,B	LMN:Mn^4+^,B,Li^+^
Space group	P2_1_/n	P2_1_/n	P2_1_/n	P2_1_/n
Symmetry	monoclinic	monoclinic	monoclinic	monoclinic
a, Å	7.960988	7.957217	7.953043	7.958700
b, Å	5.663982	5.658432	5.659001	5.655400
c, Å	5.618457	5.615722	5.617241	5.616400
V, Å^3^	252.93	252.85	253.03	252.88
Z	4	4	4	4
α = γ °	90	90	90	90
β °	89.95	89.98	89.92	89.96
R_wp_	14.6	12.6	12.1	11.3
R_p_	11.8	10.3	11.2	9.2
χ^2^	2.67	1.83	2.01	1.93

**Table 2:** Lifetimes and internal quantum efficiency values of the LMN:Mn^4+^, LMN:Mn^4+^,B, and LMN:Mn^4+^,B,R^+^ (R = Li, Na, K) phosphors.

Sample	Lifetime (ms)	α abs (%)	IQE (%)
LMN:Mn^4+^	0.847	44.2	43.3
LMN:Mn^4+^,B	0.863	58.5	50.2
LMN:Mn^4+^,B,Li^+^	0.975	67.3	61.7
LMN:Mn^4+^,B,Na^+^	0.883	60.0	52.1
LMN:Mn^4+^,B,K^+^	0.901	62.2	55.6

**Table 3:** Recent thermal stability of Mn^4+^-activated phosphors.

Sample	Thermal Stability at 423 K	E_a_ (eV)	Ref
Sr_2_LuTaO_6_:Mn^4+^	25%	0.29	[8]
SrLa_2_Al_2_O_7_:Mn^4+^	43%	0.27	[34]
CaY_0.5_Ta_0.5_O_3_:Mn^4+^	50%	0.138	[44]
CaLaLiTeO_6_:Mn^4+^	63%	0.219	[45]
Mg_3_Ga_2_SnO_8_:Mn^4+^	50%	0.255	[46]
Cs_2_NbOF_5_:Mn^4+^	61%	0.261	[47]
Li_6_SrLa_2_Sb_2_O_12_:Mn^4+^	50%	0.307	[48]
LMN:Mn^4+^	47.3%	0.149	This work
LMN:Mn^4+^,B	66.7%	0.198	This work
LMN:Mn^4+^,B,Li^+^	68.9%	0.223	This work

## Data Availability

Further research data are available from the authors on request.
